# Circular RNA Sirtuin1 represses pulmonary artery smooth muscle cell proliferation, migration and autophagy to ameliorate pulmonary hypertension via targeting microRNA-145-5p/protein kinase-B3 axis

**DOI:** 10.1080/21655979.2022.2036302

**Published:** 2022-04-03

**Authors:** Xiaogang Jing, Shujun Wu, Ying Liu, Huan Wang, QingFeng Huang

**Affiliations:** a Department of Respiratory, The First Affiliated Hospital of Zhengzhou University, Zhengzhou City, Henan Province, China; b Zhibang Biological Laboratory, Guangzhou Science City Incubation Base, Guangzhou City, Guangdong Province, 510000, China

**Keywords:** CircSIRT1, microrna-145-5p, protein kinase-B3, pulmonary hypertension, pulmonary artery smooth muscle cells

## Abstract

Recently, several studies have been clarified that circular RNA (circRNA) was a vital regulatory gene of pulmonary hypertension (PH). Nevertheless, the action of circRNA in PH was not yet explored. This study was to figure out the biological function and potential molecular mechanism of circSirtuin1 (SIRT1) in PH. Construction of the PH rat model and hypoxia pulmonary artery smooth muscle cells (PASMC) model was performed, and test of circSIRT1/microRNA (miR)-145-5p/protein kinase-B3 (Akt3) was conducted. The influence of the circSIRT1/miR-145-5p/Akt3 axis on the histopathology, hemodynamics with autophagy of the pulmonary artery in rats was examined. Additionally, the impact of circSIRT1/miR-145-5p/Akt3 on the proliferation, migration and apoptosis with autophagy of PASMC under hypoxic environment was also determined. The targeting of circSIRT1/miR-145-5p/Akt3 was testified. The results manifested that circSIRT1 and Akt3 were elevated in PH, while miR-145-5p was declined. Knockdown of circSIRT1 ameliorated rat PH, suppressed PASMC proliferation, migration with autophagy in hypoxic environment. CircSIRT1 competitively combined with miR-145-5p to mediate Akt3. To sum up, circSIRT1/miR-145-5p/Akt3 was supposed to perform as a prospective molecular target for the treatment of PH.

## Graphical abstract

**Figure:**
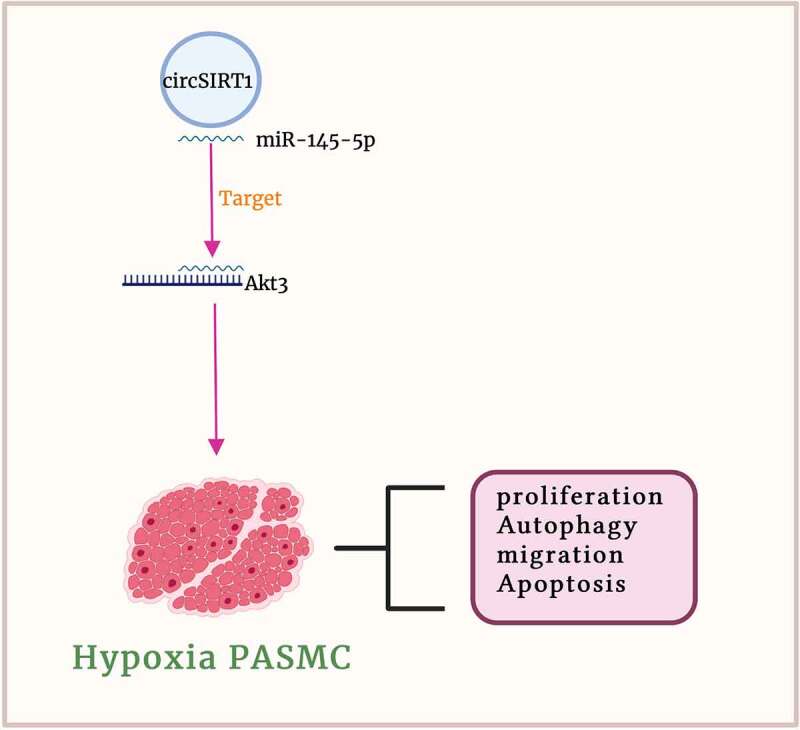

## 1 Introduction

Pulmonary hypertension (PH), a complex and lethal disease, exerted a severe threat to people’s health. PH was primarily pulmonary vascular remodeling and right ventricular hypertrophy and even failure. Pulmonary vascular remodeling was characterized via malignant proliferation of pulmonary artery smooth muscle cells (PASMC) and pulmonary artery endothelial cell dysfunction. Presently, no curative drugs were to treat PH, and lung transplantation was still a critical treatment for PH. Consequently, it is a necessity to further comprehend the underlying pathogenesis of PH, being supposed to offer new perspectives for the treatment of PH.

Circular RNA (circRNA), members of the non-coding RNA family, was actually a derivative of protein coding exons. Presently, circRNA was available to be a potential molecular target for disease diagnosis and treatment. Lately, multiple studies have partially uncovered the biological function of circRNA in PH. For instance, Wang Y *et al*. manifest that circRNA (circ) 0002062 accelerates the proliferation of PASMC via modulating the microRNA (miR)-942-5p/CDK6 axis [1]. CircHIPK3 modulates pulmonary artery endothelial cell function and blood vessel advancement via targeting the miR-328-3p/STAT3 axis [2]. Additionally, circ0068481 accelerates right ventricular hypertrophy in PH patients via modulating miR-646/miR-570/miR-885 [3]. Circ Sirtuin1 (SIRT1), a newly discovered circRNA, has been found to regulate the activation of NF-κB in vascular smooth muscle cells [4]. In addition, circSIRT1 can promote cardiac autophagy to inhibit cardiac hypertrophy [5]. A present study elucidate that 76 circRNAs are augmented in hypoxia-stimulated pulmonary hypertension rats, and 107 circRNAs are declined [6]. CircSIRT1 was elevated in PH. Nevertheless, whether circSIRT1 was involved in the development and underlying molecular mechanisms of pulmonary hypertension was yet unknown.

In this study, It was assumed that circSIRT1 participated in the development of PH. The in *vivo* and in *vitro* models of PH were constructed via monocrotaline (MCT) induction or hypoxia induction, and the biological function and downstream molecular mechanism of circSIRT1 in PH were investigated through bioinformatics prediction and functional rescue experiments.

## 2 Methods

### 2.1 Acquisition of clinical samples

From August 2015 to December 2019, lung tissue samples of Pulmonary Arterial Hypertension (PAH) were collected from 21 PAH patients who received lung transplantation in the First Affiliated Hospital of Zhengzhou University. Patients was diagnosed with PAH by echocardiography and the diagnostic procedure was in line with the European Society of Cardiology and European Respiratory Society guidelines for the diagnosis of pulmonary hypertension and pulmonary disease casued by pulmonary hypertension and left heart disease or pulmonary hypertension caused by hypoxia in 2015. Exclusion criteria: Patients suffered from a history of severe obstructive pulmonary disease, psychosis, drug addiction, or other medical conditions (chronic liver disease, portal hypertension, chronic kidney disease, amyloidosis, etc.) and had received prostacyclin, endothelin receptor antagonists, L-arginine, or sildenafil. In addition, 11 healthy lung tissue samples were taken from donors who were not suitable for transplantation. Selection criteria of the donor were as follows: <50 years old; Each year < smoking history of 20 packs of cigarettes; There was no chest trauma or continuous mechanical ventilation less than 1 week; Oxygen concentration, 1.0; Positive end-expiratory pressure, 5 cm; PaO_2_, > 300 mmHg; Relatively clear lung field in chest radiograph; Clear trachea in inspection result of Bronchoscopy. Tissue was obtained or rapidly stored at −80°C until protein extraction. This study has been approved by the Ethics Committee of the First Affiliated Hospital of Zhengzhou University, and written informed consent was presented from all subjects.

### 2.2 Construction of a rat model of PH

Purchase of forty adult male Sprague-Dawley rats (200 ± 20 g, 4–5 weeks old) was performed (Slack Jingda Animal Experiment Company, Changsha, China). The rats were kept in a pathogen-free environment with a 12 h light/dark cycle. Rats was able to have free access to standard feed and drinking water. Division of rats was into the normal and the PH (*n* = 10). Subcutaneous injection of 30 rats was with 60 mg/kg MCT (Sigma Chemicals) to stimulate pulmonary hypertension [7]. Injection of the same dose of saline was in the normal as a control. Injection of 2 × 10^8^ TU/mL sh-RNA lentiviral vector of stably targeting circSIRT1 or negative control (NC) was into the tail vein of PH rats to knock down circSIRT1 (sh-circSIRT1/NC). Euthanasia of rats was via intraperitoneal injection of 100 mg/kg sodium pentobarbital, and lung tissue samples were taken from each rat. Division of the lung tissue of each rat was into two parts, storing of one part was in 4% paraformaldehyde for subsequent pathological examination, and storing of the other was for subsequent RT-qPCR or Western blot test. Purchase of the above lentivirus plasmid vector was performed (GenePharma, Shanghai, China). Authorization of all experimental procedures was via the Animal Care Ethics and Use Committee of the First Affiliated Hospital of Zhengzhou University.

### 2.3 Hemodynamic measurement

After MCT treatment, insertion of the heparinized PV-1 catheter was into the pulmonary artery via the external jugular vein, right atrium, and right ventricle, and connection was to a pressure sensor (Statham P23ID). Adoption of a multi-function pressure meter connecting to a pressure sensor was to measure mean pumlonary artery pressure (mPAP). Measurement of systemic blood pressure (SBP) was via carotid artery cannulation. Separation of the wall of the right ventricle (RV) was from the wall of the left ventricle (LV) and the ventricular septum (IVS), and weighing was performed, separately. Thereafter, calculation of the RV hypertrophy index was via the weight ratio of the right ventricle to the left ventricle and the interventricular septum (RV/ [LV + IVS]).

### 2.4 Histopathological observation

Analysis of the pulmonary artery morphology was via hematoxylin and eosin (HE) and Masson staining. The lung tissue fixed with 4% paraformaldehyde was dehydrated, infiltrated, waxed, and embedded in paraffin for HE staining in line with standard procedures, and cut was into 4 micron sections. Subsequently, staining of the sections was with HE. Additionally, adoption of Masson staining was to test collagen deposition in pulmonary artery slices [8]. Adoption of the visual imaging system image-Pro plus 6.0 program was to capture and analyze pathological tissue imagas.

### 2.5 Immunohistochemistry (IHC)

IHC was performed as previously described [9]. Treatment of sections was with 3% H_2_O_2_ to block endogenous peroxidase activity for 10 min. Incubation of the sections was with the following antibodies Beclin-1 (3495, Cell Signaling Technology), p62 (5114, Cell Signaling Technology), ATG5 (12,994, Cell Signaling Technology). After that, addition of the secondary antibody Immunoglobulin G (IgG)was conducted (ab6785, Abcam), and incubation of the sections for another 30 min. Subsequently, after incubation with the secondary antibodies, staining of the sections was with diaminobenzidine. Block of nonspecific binding was with 5% normal goat serum for 10 min. Analysis of the IHC results was with NIH ImageJ v1.56 (National Institutes of Health, Bethesda, MD, USA).

### 2.6 Cell culture

Human PASMC was obtained (BeNa Culture Collection, Beijing, China), culture of the cells was in 90% high sugar Dulbecco’s Modified Eagle’s medium (DMEM), and placing was in the humid environment. The medium covered 10% fetal bovine serum (FBS) (Gibco, San Diego, NY, USA), 100 U/mL penicillin and 100 mg/mL streptomycin. Prior to hypoxia induction, PASMCs for 2–3 consecutive generations of PASMCs were starved in serum-free DMEM medium for 24 h. Subsequently, hypoxia induction was conducted, and culture of the PASMC was for 48 h under hypoxic conditions.

### 2.7 Cell transfection

Small interfering RNA of targeting circSITR1 and protein kinase-B3 (Akt3) (si-circSITR1 and si-Akt3) and elevating plasmids (pcDNA 3.1-circSIRT1 and pcDNA 3.1-Akt3) and NC (si-NC or pcDNA 3.1) were designed and provided (Shanghai Gene Pharmaceutical Co., Ltd., Shanghai, China). Purchase of miR-145-5p mimic/inhibitor and mimic/inhibitor NC were conducted (GenePharma, Shanghai, China). Transfection of the plasmids or oligonucleotides was into PASMC adopting Lipofectamine 2000 transfection reagent (Invitrogen, CA, USA) in line with the manufacturer’s methods. After transfection of 48 h, test of the transfection efficiency was performed.

### 2.8 Edu staining is to test proliferation

Edu experiment was performed as previously described [10]. EdU staining was performed adopting Click iT™ EdU Cell Proliferation Detection Kit (Molecular Probes, Invitrogen). Seeding of the cells was into a 96-well plate, and staining was with 50 μM EdU for 2 h. After that, fixation was with 50 μL fixative (PBS + 4% polyoxyethylene), and incubation was for 30 min. Ultimately, discoloration of the cells 2–3 times was with 100 μL penetrant (PBS + 0.5% TritonX-100) (rinse for 10 min each time). Staining of the nuclei was with 4’, 6-diamidino-2-phenylindole for 10 min. Examination of the results of cell staining was with a fluorescence microscope (Olympus, Tokyo, Japan).

### 2.9 Test of cell apoptosis is via Flow cytometry

Assessment of the cell apoptosis was performed adopting the annexin V- fluoresceinisothiocyanat/propidium iodide (PI) apoptosis kit (BD Biosciences, CA, USA). Digestion of the transfected PAMSCs was with trypsin, obtainment was performed, and collection was conducted after centrifugation. Staining was with Annexin V and PI in line with the instructions. Examination of the apoptotic cells was performed adopting Flow cytometry (BD Bioscience) and Cell Quest Pro software.

### 2.10 Wound healing testing

Cell migration was detected by scratch assay [11]. Culture of PASMC in a 24-well plate was in DMEM supplemented with 10% FBS for 24 h until the cells were 80–90% confluence. The linear wound trajectory was generated with a sterile 1 mL pipette and kept under standard conditions. Rinse of the streaked cells twice with sterile PBS was to remove non-adherent cells, and addition of 0.2% FBS was in DMEM. The image of the center of the gap was taken with an optical microscope. Assessment of cell migration at 0 and 24 h after the scratches was via determining the wound distance at two random wound gap positions.

### 2.11 Reverse transcription quantitative polymerase chain reaction (RT-qPCR)

RT-qPCR was performed in line with the foregoing procedure. Extraction of total RNA was from tissues or PASMCs with Trizol reagent (AP-MN-MS-RNA-50, Axygen, USA) in line with the manufacturer’s instructions. Reverse transcription of RNA was into a complementary DNA (cDNA) adopting the Prime-Script RT kit (RR047A, Takara, Japan). Amplification of cDNA was in LightCycler480 real-time PCR system (Applied Biosystems, Foster City, CA) adopting SYBR Premix Ex Taq™ II (RR820A, Takara, Japan). Normalization of target gene was via 2^−ΔΔCT^ method. Adoption of glyceraldehyde-3-phosphate dehydrogenase (GAPDH) and U6 was as internal reference genes for mRNA and miRNA, separately. The primer sequence is presented in Table 1.

**Table 1.** RT-qPCR primer sequences

	Primer sequences (5′ – 3′)
GAPDH	Forward: 5’-ATCTTCCAGGAGCGAGATCCC-3’
	Reverse: 5’-TGAGTCCTTCCACGATACCAA-3’
U6	Forward: 5’- GCTTCGGCAGCACATATACTAAAAT −3’
	Reverse: 5’- CGCTTCACGAATTTGCGTGTCAT −3’
MiR-145-5p	Forward: 5’- CTCACGGTCCAGTTTTCCCA −3’
	Reverse: 5’- ACCTCAAGAACAGTATTTCCAGG −3’
Akt3	Forward: 5’- CTGAGGACCGCACACGTTTCTA −3’
	Reverse: 5’- TGGCCATCTTTGTCCAGCATTA −3’
CircSIRT1	Forward: 5’-AGAGATTGTGTTTTTTGGTGAA-3’
	Reverse: 5’- GAAGGTTATTTGGAATTAGTGC −3’

### 2.12 Western blot

Extraction of proteins was from tissues and cells adopting Radio-Immunoprecipitation assay lysis buffer. Quantification of protein concentration was performed adopting a bicinchoninic acid kit (Nanjing KGI Biotechnology Co., Ltd., Nanjing, China). Separation of total protein (20 g) was via 10% sulfate polyacrylamide gel electropheresis, and electroblot was onto nitrocellulose membrane, which was incubated with the following specific antibodies: Beclin-1 (3495, Cell Signaling Technology), p62 (5114, Cell Signaling Technology), ATG5 (12,994, Cell Signaling Technology), GAPDH (2118, Cell Signaling Technology, Akt3 (9272, Cell Signaling Technology), Akt1(sc-5298, Santa Cruz Biotechnology), Akt2 (3063, Cell Signaling Technology), LC3B (2775, Cell Signaling Technology). Subsequently, incubation of the membrane was with goat anti-rabbit IgG secondary antibody (ab6785, Abcam). Imaging was performed adopting electrogenerated chemiluminescence plus detection reagent (Thermo Fisher Scientific, Inc.). Analysis of the imprinted density was via ImageJ software.

### 2.13 The luciferase reporter experiment

Association of miR-145-5p withcircSIRT1 or Akt3 was predicted adopting bioprediction websites. Purchase of circSIRT1 or Akt3 3ʹuntranslated region wild-type (WT) or mutant (MUT) pmirGLO dual-luciferase vector covering the predicted miR-145-5p binding site was performed (Shanghai Gene Pharmaceutical Co., Ltd., Shanghai, China). Co-transfection of the plasmids and miR-145-5p mimic or mimic NC was into PASMC adopting Lipofectamine™ 2000 transfection reagent (Invitrogen, CA, USA) in line with the manufacturer’s requirement. Examination of the luciferase activity was with a luciferase reporter gene test kit (E1910, Promega, Madison, WI, USA). Measurement of the fluorescence intensity was with a GloMax 20/20 fluorescence detector (E5311, Shaanxi Sino-American Biotechnology Co., Ltd., Shanxi, China) (*N* = 3).

### 2.14 Statistical analysis

Representation of the data was as mean ± standard deviation (SD). Selection of student t test was to compare the differences between the two. Adoption of one-way analysis of variance (ANOVA) and Tukey’s post-hoc test was for multi-group comparisons. All statistical analyses were performed adopting GraphPad Prism 9 software (La Jolla, CA). All experimental groups covered at least three biological replicates. *P* < 0.05 was accepted as indicative of significant differences.

## 3 Results

### 3.1 CircSIRT1 is elevated in pulmonary hypertension rats

**Figure 1.**
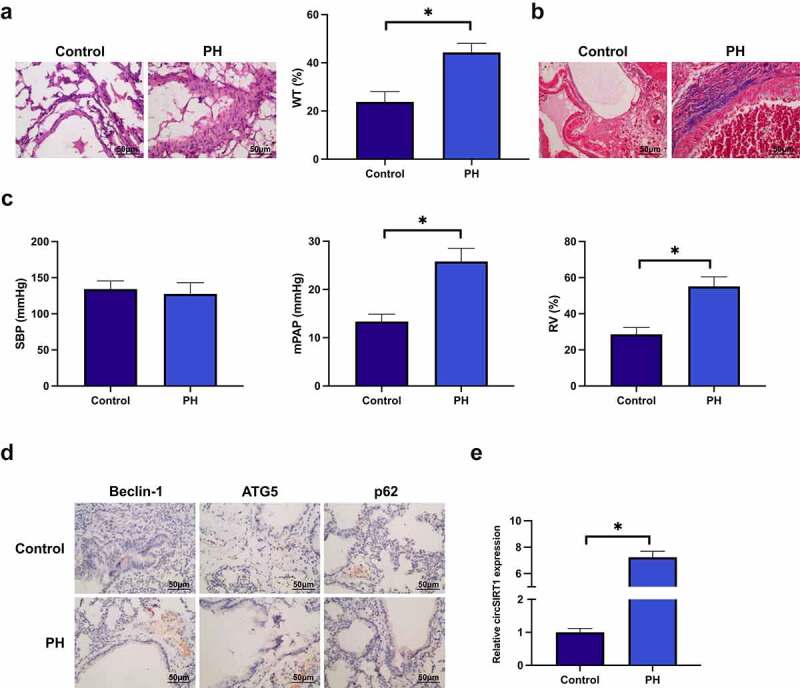
CircSIRT1 is elevated in rats with pulmonary hypertension. A: Representative image of HE staining of rat pulmonary artery after MCT induction; B: Representative image of Masson staining of rat pulmonary artery after MCT induction; C: Hemodynamic index of rats after MCT induction; D: Immunohistochemistry test of MCT induction influence of MCT induction on Beclin-1, ATG5 and p62 in rat pulmonary artery; E: RT-qPCR test of the impact of MCT induction on circSIRT1 in rat pulmonary artery; Representation of data was as mean ± SD (*n* = 10); *P < 0.05.

Construction of a PH model was via MCT induction to explore the biological function of circSIRT1 in pulmonary hypertension. The thickness of pulmonary artery wall in rats was distinctively elevated after MCT induction (Figure 1A). MCT induction augmented collagen deposition in rat pulmonary arteries (Figure 1B). Additionally, hemodynamic measurements were performed on rats. The results clarified that MCT induction elevated mPAP and RVI index in rats, but no distinct influences were presented on SBP (Figure 1C). Subsequently, examination of autophagy-correlated proteins was via immunohistochemistry. MCT induction accelerated pulmonary artery Beclin-1 and ATG5, but it suppressed p62, as proved in Figure 1D. After MCT induction, circSIRT1 in rat lung tissue was critically elevated (Figure 1E). These data manifested that a PH model was successfully constructed, and circSIRT1 was elevated in PH.

### 3.2 Knockdown of circSIRT1 ameliorates PH

**Figure 2.**
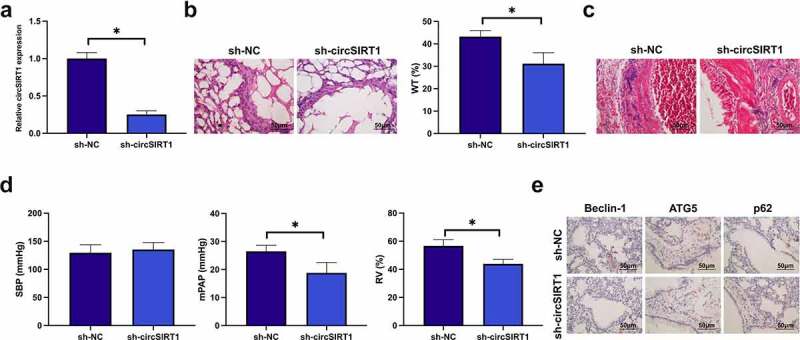
Knockdown of circSIRT1 ameliorates PH. A: RT-qPCR test of circSIRT1 in the sh-NC and SH-CircSIRT1; B: Representative images of HE staining of rat pulmonary artery after knockdown of circSIRT1; C: Representative images of Masson staining of rat pulmonary artery after knockdown of circSIRT1; D: Hemodynamic indexes of rats after knockdown of circSIRT1; E: Immunohistochemical detection of the influence of knockdown of circSIRT1 on Beclin-1, ATG5 and P62 in rat pulmonary artery; Representation of data was as mean ± SD (*n* = 10); *P < 0.05.

Injection of sh-circSIRT1 lentiviral vector was into PH rats to knock down circSIRT1. Sh-circSIRT1 distinctively declined circSIRT1 in PH rats, as proved in Figure 2A. Subsequently, histopathological examination was performed. Knockdown of circSIRT1 declined the thickness of the pulmonary artery wall and mitigated the degree of pulmonary artery fibrosis, as proved in Figure 2B, C. Hemodynamic data elucidated that knockdown of circSIRT1 declined mPAP and RVI indexes (Figure 2D). IHC results clarified that knockdown of circSIRT1 restrained Beclin-1 and ATG5 and augmented P62 in pulmonary arteries (Figure 2E). In general, knockdown of circSIRT1 effectively ameliorated PH.

### 3.3 Knockdown of circSIRT1 suppresses hypoxia-stimulated PASMC cell advancement with autophagy

**Figure 3.**
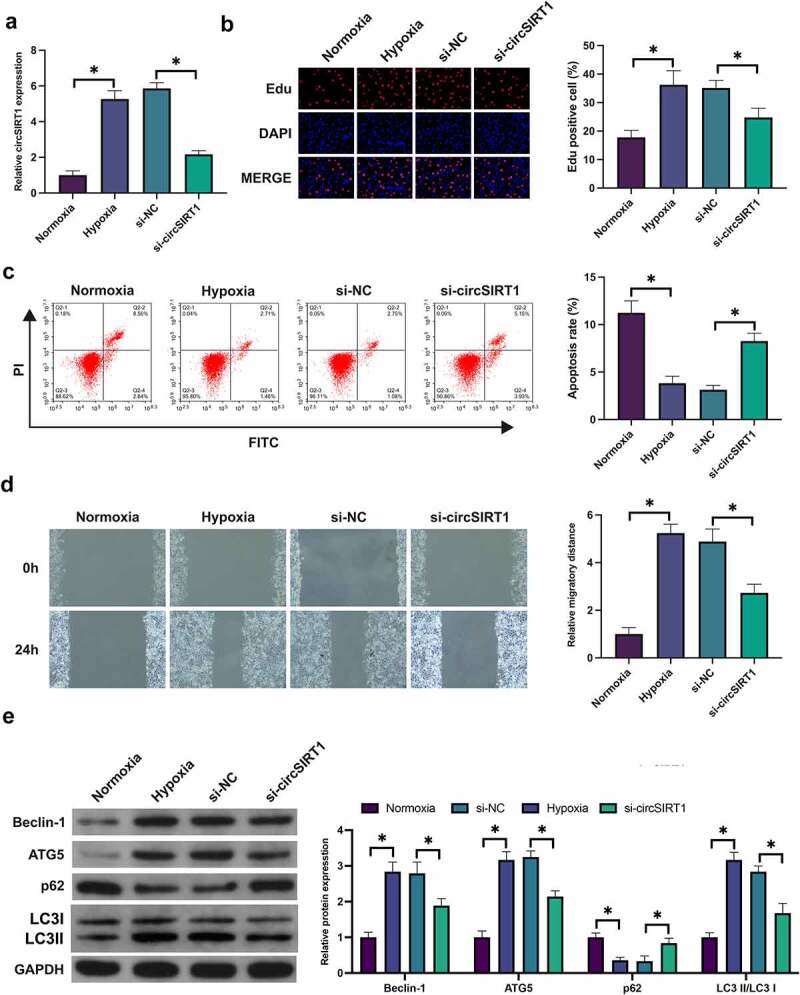
Knockdown of circSIRT1 suppresses hypoxia-stimulated PASMC cell advancement with autophagy. A: RT-qPCR test of circSIRT1 in PASMC after hypoxia-induction or knockdown of circSIRT1; B: Edu staining examination of PASMC proliferation after hypoxia-induction or knockdown of circSIRT1; C: Flow cytometry test of apoptosis of PASMC after hypoxia-induction or knockdown of circSIRT1; D: Scratch test examination of PASMC migration after hypoxia-induction or knockdown of circSIRT1; E: Western blot test of Beclin-1, ATG5 and p62 in PASMC after hypoxia-induction or knockdown of circSIRT1; Representation of data was as Mean ± SD (*n* = 3); *P < 0.05.

*In vitro* experiments were conducted to further explore the action of circSIRT1 in PH. Construction of a *in vitro* PH model was via hypoxia-stimulated PASMC. Hypoxia induction elevated circSIRT1 in PASMC, while transfection with si-circSIRT1 declined circSIRT1, as presented in Figure 3A. The proportion of Edu-positive cells was elevated after hypoxia induction, but knockdown of circSIRT1 declined the proportion of Edu-positive cells (Figure 3B). Hypoxia induction declined the apoptosis rate of PASMC, but knockdown of circSIRT1 augmented it (Figure 3C). Hypoxia induction augmented the migration of PASMC, but knockdown of circSIRT1 declined it (Figure 3D). Additionally, beclin-1 and ATG5 in PASMC were elevated, while p62 was declined after hypoxia induction, but knockdown of circSIRT1 prevented this phenomenon (Figure 3E). In brief, knockdown of circSIRT1 repressed hypoxia-stimulated PASMC cell advancement with posture.

### 3.4 Circ_ANKIB1 competitively combines with miR-26b-5p

**Figure 4.**
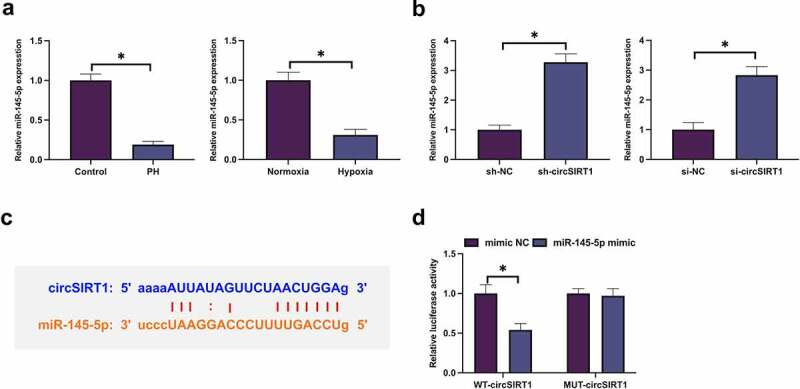
CircSIRT1 competitively combines with miR-26b-5p. A: RT-qPCR examination of miR-145-5p in PH rats or hypoxia-stimulated PASMC; B: RT-qPCR test of miR-145-5p in rats or PASMC after knockdown of circSIRT1; C: Bioinformatics website http://starbase.sysu.edu.cn/ prediction of circSIRT1 and miR-145-5p; D: The dual luciferase report experiment verification of the targeting of circSIRT1 with miR-145-5p; Representation of the data was as the mean ± SD (*n* = 3/10); Representation of the data was as the mean ± SD (*n* = 3); *P < 0.05.

CircRNA frequently performed as a miRNA sponge and mediated downstream target genes to realize its biological functions. Subsequently, the target genes of circSIRT1 were explored. MiR-145-5p was declined in PH rats and hypoxia-stimulated PASMC (Figure 4A). Additionally, Knockdown of circSIRT1 significantly promoted the expression of miR-145-5p in tissues and cells (Figure 4B). A speculation that circSIRT1 was supposed to target miR-145-5p. CircSIRT1 was provided with feasible binding sites with miR-145-5p (Figure 4C). Subsequently, their targeting was further examined. As presented in Figure 4D, co-transfection of WT-circSIRT1 and miR-145-5p mimic into PASMC declined luciferase activity, but no distinct alteration was presented in luciferase activity after co-transfection of MUT-circSIRT1 and miR-145-5p mimic into PASMC. In short, circSIRT1 competitively combined with miR-145-5p.

### 3.5 CircSIRT1 impacts the biological behavior of hypoxia-stimulated PASMC via modulating miR-145-5p

**Figure 5.**
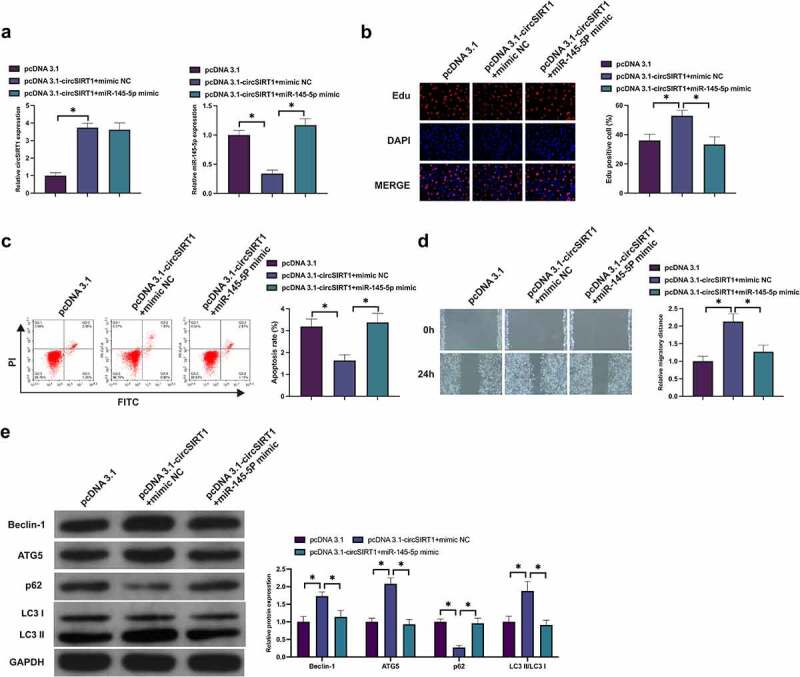
CircSIRT1 impacts the biological behavior of hypoxia-stimulated PASMC via modulating miR-145-5p. A: Test of circSIRT1 and miR-145-5p in PASMC after co-transfection of pcDNA 3.1-circSIRT1 and miR-145-5p mimic was via RT-qPCR; B: Examination of PASMC proliferation after co-transfection of pcDNA 3.1-circSIRT1 and miR-145-5p mimic was via Edu staining; C: Detection of PASMC apoptosis after co-transfection of pcDNA 3.1-circSIRT1 and miR-145-5p mimic was via Flow cytometry; D: Examination of PASMC migration after co-transfection of pcDNA 3.1-circSIRT1 and miR-145-5p mimic was via Scratch test; E: Detection of Beclin-1, ATG5 and p62 in PASMC after co-transfection with pcDNA 3.1-circSIRT1 and miR-145-5p mimic was via Western blot; Representation of data was as mean ± SD (*n* = 3); *P < 0.05.

Whether miR-145-5p was involved in circSIRT1 to modulate PASMC was examined. As presented in Figure 5A, transfection of pcDNA 3.1-circSIRT1 elevated circSIRT1 in hypoxia-stimulated PASMC, but miR-145-5p was declined, while co-transfection with miR-145-5p mimic turned around this phenomenon. Elevation of circSIRT1 accelerated the Edu positive rate of PASMC, but augmentation of miR-145-5p prevented this action (Figure 5B). Elevation of circSIRT1 boosted PASMC advancement, while augmentation of miR-145-5p recovered PASMC’s progression (Figure 5C, D). Elevation of circSIRT1 promoted the migration ability of PASMC, while elevation of miR-145-5p at the same time declined the migration rate of PASMC (Figure 5D). Additionally, elevation of circSIRT1 augmented Beclin-1 and ATG5 in PASMC, and p62 was declined, while augmentation of miR-145-5p turned around the proteins (Figure 5E). These data clarified that circSIRT1 influenced hypoxia-stimulated PASMC advancement with autophagy via modulating miR-145-5p.

### 3.6 MiR-145-5p targets Akt3

**Figure 6.**
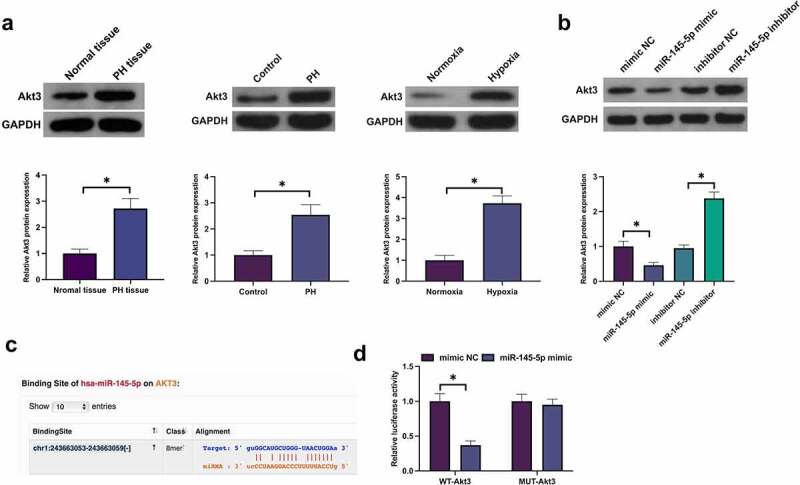
MiR-145-5p targets Akt3. A: Examination of Akt3 in PH tissue or hypoxia-stimulated PASMC was via Western blot; B: Test of Akt3 in hypoxia-stimulated PASMC after knockdown or elevation of miR-145-5p was via Western blot; C: Prediction of Akt3 and miR-145-5p was via bioinformatics website http://starbase.sysu.edu.cn/http://starbase.sysu.edu.cn/ Forecast Akt3 and miR-145-5p D: Verification of the targeting of Akt3 with miR-145-5p was via the dual luciferase report experiment; Representation of the data was as the mean ± SD (*n* = 3/10); *P < 0.05.

Target gene of miR-145-5p was further figured out. Akt3 expression was significantly increased in PH tissues and hypoxia-induced PASMC (Figure 6A). Additionally, elevation of miR-145-5p or knockdown of miR-145-5p repressed and facilitated Akt3, respectively (Figure 6B). Bioinformatics website predicted that miR-145-5p was provided with feasible binding sites with Akt3 (Figure 6C). Subsequently, the targeting of miR-145-5p with Akt3 was verified. The results manifested that luciferase activity was declined after co-transfection of WT-Akt3 and miR-145-5p mimic into PASMC, but co-transfection of MUT-Akt3 and miR-145-5p mimic exerted no influence on luciferase activity of PASMC. In short, miR-145-5p targeted Akt3.

### 3.7 CircSIRT1 impacts the biological behavior of hypoxia-stimulated PASMC via modulating miR-145-5p/Akt3 pathway

**Figure 7.**
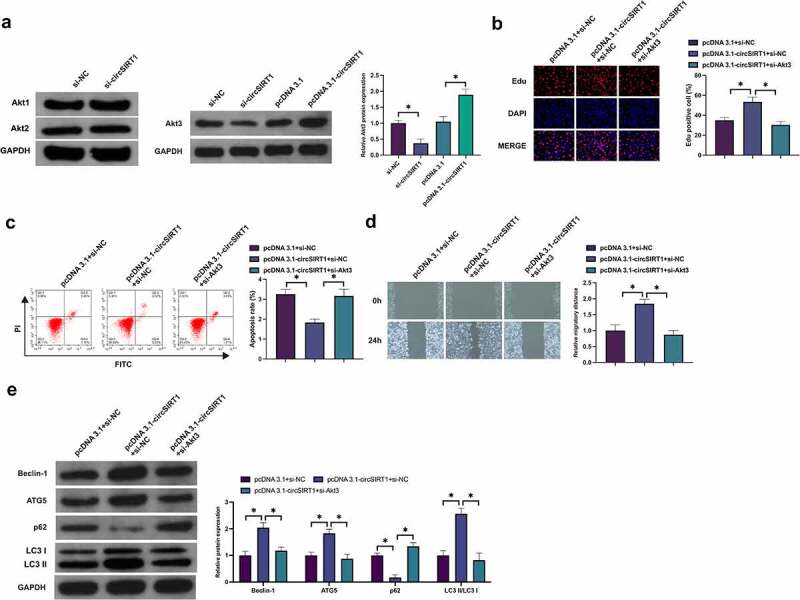
CircSIRT1 impacts the biological behavior of hypoxia-stimulated PASMC via modulating miR-145-5p/Akt3 pathway. A: Test of Akt1-3 in PASMC after knockdown or elevation of circSIRT1 was via Western blot; B: Examination of PASMC proliferation after co-transfection of pcDNA 3.1-circSIRT1 and si-Akt3 was via Edu staining; C: Detection of PASMC apoptosis after co-transfection of pcDNA 3.1-circSIRT1 and si-Akt3 was via Flow cytometry; D: Examination of PASMC migration after co-transfection of pcDNA 3.1-circSIRT1 and si-Akt3 was via Scratch test; E: Detection of Beclin-1, ATG5 and p62 in PASMC after co-transfection with pcDNA 3.1-circSIRT1 and si-Akt3 was via Western blot; Representation of data was as mean ± SD (*n* = 3); *P < 0.05.

Whether circSRIT1 was able to exert an influence on the biological behavior of hypoxia-stimulated PASMC via modulating the miR-145-5p/Akt3 axis was figured out. Knockdown of circSIRT1 or overexpression of circSIRT1 inhibited and promoted Akt3 expression, respectively, while knockdown of circSIRT1 had no effect on Akt1 and Akt2 expression (Figure 7A). Subsequently, co-transfection of pcDNA 3.1-circSIRT1 and si-Akt3 was into hypoxia-stimulated PASMC. As presented in Figure 7B, transfection with pcDNA3.1-circSIRT1 boosted Akt3, and miR-145-5p was declined, but this action was turned around via co-transfection with si-Akt3. Transfection with pcDNA 3.1-circSIRT1 elevated the Edu positive rate and advancement with autophagy of PASMC, but these roles were turned around via co-transfection with si-Akt3 (Figure 7C-E). In brief, circSRIT1 exerted an influence on the biological behavior of hypoxia-stimulated PASMC via modulating the miR-145-5p/Akt3 axis.

## 4 Discussion

Pulmonary hypertension is a multifactorial pathological process, involving the malignant advancement of PASMC [12,13]. It is a necessity to determine the appropriate treatment target for pulmonary hypertension. In this study, knockdown of circSIRT1 was conducive to ameliorating the hemodynamic index of PH rats, declining pulmonary artery wall thickness and fibrosis, and repressing autophagy. *In vitro* studies clarified that knockdown of circSIRT1 suppressed hypoxia-stimulated PASMC advancement with autophagy via targeting miR-145-5p/Akt3 axis.

The signature features of PH are elevated blood vessel wall thickness and pulmonary artery fibrosis [14]. Foregoing studies have manifested that knockdown of circ0000790 declines the thickness of the pulmonary artery wall in PH rats [15]. In this study, knockdown of circSIRT1 was provided with analogical actions. Pulmonary artery fibrosis was manifested as the elevation of vascular fibers and connective tissue and the decline of parenchymal cells, leading to the destruction of organ structure and vascular sclerosis. Multiple studies have elucidated that circRNA is involved in the progression of pulmonary fibrosis, covering circ0026344 [16], circ0000981 [17] and circ0044226 [18]. In this study, circRNA-SIRT1 was involved in the degree of pulmonary artery wall, which was supposed to have a critical association with pulmonary artery remodeling. Autophagy is the highly modulated cell catabolic process, involving the degradation of solute components of the cells (covering dysfunctional organelles and misfolded proteins) [19,20]. Autophagy is usually activated under stress conditions like hypoxia, inflammation and reactive oxygen species [21]. The activation of PAMSC autophagy is conducive to offering sufficient energy to its aberrant proliferation during hypoxia [22,23]. A great deal of studies has manifested that circRNA exerts a vital role in modulating cell autophagy. The latest study has elucidated that circ0030042 modulates aberrant autophagy of endothelial cells via targeting eIF4A3 to protect the stability of the atherosclerotic plate [24]. Additionally, Wei M *et al*. maintain that circHIPK3 ameliorates atherosclerosis via modulating endothelial cell autophagy [25]. In this study, circSIRT1 declined hypoxia-stimulated PAMSC autophagy, which was supposed to have a critical association with its suppression of PAMSC’s aberrant advancement, seeing that the repression of autophagy declined the energy source for PAMSC’s progression.

To further comprehend the molecular mechanism of circSIRT1 in PH, its miRNA and downstream target genes of adsorption was explored. CircSIRT1 is a circular structure, which is stably expressed in the body and not easily degraded. Mechanically, circSIRT1, as a ceRNA, plays a role of miRNA sponge in the body due to its rich binding sites of miR-145-5p in the closed loop structure, and the inhibitory effect of miR-145-5p on Akt3 is competitively removed, thus up-regulating Akt3 expression. Furthermore, Akt3 is regulated at the transcriptional level.A present study reported the influence of miR-145-3p on cancer autophagy. MiR-145-5p elevated the autophagy flux of laryngeal squamous cell carcinoma via activating the Akt/mTOR pathway to suppress cancer advancement [26]. Previous studies have clarified that miR-145-5p represses the progression, oxidative stress and local inflammation of vascular smooth muscle cells via targeting SMAD4, FGF10 or EGR1 [27,28]. In this study, miR-145-5p repressed PAMSC advancement with autophagy flux via targeting Akt3, thereby ameliorating PH’s symptoms. Notably, the Akt3/mTOR pathway is a vital molecular target for the modulation of intracellular autophagy [29–31]. The alteration of multiple intracellular and extracellular signals is able to be sensed [32]. Additionally, the speed of autophagy is available to be activated or repressed [33,34]. In this study, augmentation of circSIRT1 on the advancement with autophagy of PAMSC was rescued via knocking down Akt3.

## 5 Conclusion

In short, knockdown of circSIRT1 is conducive to ameliorating PH, repressing PAMSC advancement with autophagy. Mechanistically, circSIRT1 competitively combined with miR-145-5p and mediated Akt3 to exert its role. Furthermore, the potential of circSIRT1/miR-145-5p/Akt3 was identified to perform as a target site for PH treatment. Nevertheless, more clinical data were needed to support the results of this study, which required to be explored in subsequent studies.
